# Co-Design of a Physical Activity Maintenance Intervention for People With Stroke: Protocol for a Mixed Methods Study

**DOI:** 10.2196/89913

**Published:** 2026-05-06

**Authors:** Katy Pedlow, Suzanne McDonough, Natalie Duff, Karen McConnell, Claire McFeeters, Angela Carlin, Noelene Hughes, Zoe Campbell, Andrew Bell, Aoife Stephenson, Gary Adamson, Jemma Hawkins, Sarah Howes

**Affiliations:** 1 School of Health Sciences, University of Ulster, Northland Rd, Londonderry, Northern Ireland, BT48 7JL, United Kingdom, 44 02871675942; 2 Royal College of Surgeons in Ireland, Dublin, Ireland; 3 School of Nursing and Midwifery, Queen's University Belfast, Belfast, Northern Ireland, United Kingdom; 4 Centre for Exercise Medicine, Physical Activity and Health, Sports and Exercise Sciences Research Institute, University of Ulster, Belfast, Northern Ireland, United Kingdom; 5 Northern Ireland Chest Heart and Stroke, Belfast, Northern Ireland, United Kingdom; 6 School of Psychology, University of Ulster, Coleraine, TX, United Kingdom; 7 DECIPHer, School of Social Sciences, Cardiff University, Cardiff, Wales, United Kingdom

**Keywords:** physical activity maintenance, stroke, postrehabilitation, adults, fitness professionals, co-design

## Abstract

**Background:**

Stroke is a global health problem that often causes physical disability and mental health issues for the survivor. While physical activity (PA) improves outcomes post stroke, it can be challenging to maintain. Barriers to maintaining PA post stroke include the setting of PA, motivation, and impairments from the stroke. There is often a desire to maintain PA after stroke, but effective interventions are currently limited.

**Objective:**

This study aims to coproduce an intervention to support long-term PA maintenance for adults living with the effects of stroke in Northern Ireland. The objectives of this study are to understand the perspectives of key stakeholders on the components, structure, and content of an intervention to support PA maintenance and to coproduce and refine a prototype intervention to meet their specific needs and preferences.

**Methods:**

A mixed methods study will be conducted, consisting of three stages informed by the Centre for Development, Evaluation, Complexity and Implementation in Public Health Improvement (DECIPHer) co-production framework. Stage 1 will include a scoping review on PA maintenance in survivors of stroke and stakeholder consultation via focus groups to gain understanding from their perspective of PA. Survivors of stroke and their carers, physiotherapists, and care coordinators from community and voluntary organizations will be recruited from ongoing Post Rehabilitation Exercise Program (PREP) classes. Additional health care professionals with experience in PA and stroke will also be recruited via relevant organizations. Individuals who complete stage 1 focus groups will be invited to take part in stage 2 co-design workshops to develop a PA maintenance program for participants post PREP. Stage 3 will involve expert review of the co-production program by members of the project advisory board via a questionnaire survey. Qualitative data will be analyzed using reflexive thematic analysis from data collected in stages 1 and 2. Data from the scoping review will help shape the questions for the focus groups, and data from the focus groups will help inform questions for the three workshops. All stages will involve the stakeholders to gain feedback and suggestions for the next wave.

**Results:**

Five focus groups were conducted with 38 participants: three in-person (stroke survivors and their caregivers) and two online (one for PREP staff and one for other health and exercise professionals) between September and November 2025. Results from the focus groups identified two themes: shifting realities of physical activity post stroke and motivation through community. Eighteen participants subsequently consented to participate in three co-design workshops, which resulted in an intervention prototype being developed.

**Conclusions:**

This study aims to co-develop an intervention to support PA maintenance in adults living with stroke after completion of community rehabilitation. To our knowledge, there is no further support for the survivors to help maintain their PA levels once they finish the 6- to 12-week community program. Engaging with survivors of stroke and their carers, PREP staff, and other exercise professionals will help shape the beginning stages of this study. Upcoming results from the pilot study will provide vital information on how to support PA maintenance in this population.

## Introduction

### Background

Stroke is a significant global health issue, ranked as the third-leading cause of disability [1]. Each year, approximately 12.2 million individuals are affected by stroke worldwide [2]. The impact of stroke goes beyond its burden on health care systems; it often results in individuals not fully regaining their previous level of functioning in everyday life [3]. Promoting and maintaining regular physical activity (PA) remains a major challenge worldwide, particularly for stroke survivors, as their levels of daily PA tend to be significantly lower than those of the general population [4]. PA is defined as any movement the body makes that results in an increase in energy expenditure, for instance, walking the dog or doing household chores [4,5]. Exercise is defined as any PA that is often planned and structured with a goal of maintaining/improving one’s PA level, for instance, swimming or going for a run [4,5]. Daily PA, including exercise, has been widely recognized as a crucial factor in the recovery process for individuals who have experienced stroke [6,7]. This helps to mitigate the physical and psychological impairments associated with stroke and plays a vital role in preventing future strokes [8]. Despite the rights of people with disabilities, such as stroke, to full participation and inclusion in their communities [9], there are complex and multifaceted barriers [10,11]. A recent patient and public involvement survey from a Post Rehabilitation Exercise Program (PREP) identified that participants had a desire to continue engaging in long-term PA. Currently, health care professionals can refer people to local gym settings via PA referral schemes [12]; however, within Northern Ireland, people with stroke are not eligible to participate in such schemes based solely on their stroke diagnosis [12-15]. Additionally, barriers such as lack of knowledge, confidence, limited access, and insufficient support in a gym setting hindered their ability to continue.

Regular PA, which involves being consistently active over time, is essential for long-term health benefits [16]. While there is a lack of research specifically involving the stroke population [17-19], a review in 2014 [20] suggested that any intervention content should incorporate behavior change techniques to enhance the success of PA maintenance [20-25]. A further consideration is the environment of such interventions. The European Stroke Action Plan 2018-2030 [26] highlights the need to offer PA programs to all stroke survivors living in the community. Supporting research also outlines the desire from stroke survivors to participate in longer-term programs that are community based and delivered by exercise professionals rather than being guided by a physiotherapist within a health care setting [27]. Most studies focusing on the stroke population have considered how to support adherence within structured exercise programs [28,29]. These interventions are short-term; often 6 weeks in length; and, therefore, do not consider strategies to support people with stroke to engage in PA in the longer term [30].

One strategy to support stroke survivors’ engagement in PA is the implementation of programs led by third sector organizations, such as PREP, delivered by Northern Ireland Chest Heart and Stroke (NICHS) [31]. PREP is a successful physiotherapist-led exercise program that takes place once a week for a 6- to 12-week course. This exercise program also includes a social opportunity and education sessions focused on health and well-being [32]. Evaluations of PREP have shown that this is a successful program that has helped several survivors of stroke by improving their physical function, self-confidence, and quality of life [32,33]. Although successful in providing group-based exercise in the short-term, there is currently no provision for the longer-term support to remain active once programs are completed. One way to fill this gap could be via continued partnerships between health care and voluntary/community sectors [34].

### Aim and Objectives of Study

The overall aim of the study is to develop a PA maintenance intervention for those living with stroke. The first objective is to gather information from key stakeholders, that is, patients, carers (ie, family and friends), and health care professionals, about the requirements and preferences for an intervention. The second objective is to coproduce and refine a PA maintenance intervention for people with stroke. Bringing stakeholders together as active and equal partners in health research has been highlighted to support the development of interventions that are relevant and acceptable and more likely to be implemented in health settings [35].

By adopting the Centre for Development, Evaluation, Complexity and Implementation in Public Health Improvement (DECIPHer) framework for the co-production and prototyping of interventions [36], the study will ensure the active involvement and participation of relevant stakeholders in the design and refinement of services and interventions [37].

The study will be supported by an independent project advisory group that has been created with representation across several stakeholder groups, including people with stroke, their carers, health and exercise professionals, researchers, and staff from a voluntary sector organization (NICHS).

## Methods

### Study Design

A mixed methods study will be undertaken involving three stages of the DECIPHer framework for the co-design and prototyping of interventions [36]. The study is registered with ClinicalTrials.gov, but due to restrictions in government funding, we are awaiting the trial registration number.

The framework consists of three stages:

1. Evidence review and stakeholder consultation (study objective 1): A scoping review (in progress) and stakeholder consultation (using focus group methodology) will identify potential content and structures for a PA maintenance intervention.
2. Co-design (study objective 2): A series of co-design workshops involving key stakeholders will aim to create an intervention to support long-term PA post stroke.
3. Prototype development (study objective 2): An expert review of the coproduced intervention and materials will be conducted.

### Patient and Public Involvement Statement

On the advisory board, an adult living with stroke who has previously completed PREP was involved in the study design and will continue to be involved throughout the study. They will be remunerated for their time and will be named in publications related to the study. While having one participant may be seen as a limitation, extensive patient and public involvement was completed before the development of this proposal. This included engaging with over 30 current participants of PREP. Furthermore, the co-design aspect of this study focuses on the involvement of people who are key to the intervention development.

### Evidence Review and Stakeholder Consultation (Stage 1)

A scoping review is currently being completed to summarize evidence on the content and structure of community-based PA maintenance programs for adults post stroke. This review will be conducted based on 6 studies [38-43] that looked at different PA programs for survivors of stroke. These studies looked at interventions longer than 3 months, behavior change techniques, and a variety of PA [38-43]. Through this review, our aim is to analyze, synthesize, and then combine current literature that identifies different behavior change techniques, intervention types, and characteristics. We also aim to understand the experience of those living with stroke and their experience partaking in these interventions for more than 3 months. This review will allow us to use the previous literature to help build focus group questions such as their experience of engaging in PA pre- and poststroke and gain an understanding of a PA intervention that they would like to see in the future. This review will also help in developing the structure of topics within the workshops by combining what was said in the focus groups and the main points from the literature, such as the settings of an intervention, instructors, and barriers that they may experience.

A series of focus groups will be used to understand stakeholder perspectives and ideas on the content and structure of a PA maintenance intervention for people with stroke. Focus group questions will be coproduced with the project advisory group and informed by the scoping review results. The focus group methodology will allow for in-depth insights from a range of stakeholders [44]. During this stage, stakeholders will include people with stroke who have completed PREP, their carers, and professionals working in PA for stroke such as physiotherapists, exercise professionals, and statutory or voluntary organization staff. Using a range of stakeholders will ensure the developed intervention fits the holistic needs of the target population and the wider setting the intervention will sit within.

### Inclusion and Exclusion Criteria

Textbox 1 outlines the inclusion and exclusion criteria for the three stakeholder groups that are taking part in the study.

Textbox 1. **Inclusion and exclusion criteria of each of the main groups involved.**

**Focus group (Post Rehabilitation Exercise Program [PREP] participants)**

Inclusion criteria

- Adults 18 years and older with a clinical diagnosis of stroke, who are currently attending or have completed PREP. Adults with communication impairment will be able to participate via alternative methods of communication (eg, assistive communication device). Participants who require translation services can participate if they have access to this support.
- Carers who have supported an adult who has a clinical diagnosis of stroke in completing PREP, either currently or who have already completed PREP

Exclusion criteria

- Unable to participate in a group-based conversation due to impairments
- Carers who have no knowledge or experience of PREP
- Unable to use/access a digital device with internet access and a microphone (for online focus group only)
- Being a member of the advisory board

**Focus group (professionals)**

Inclusion criteria

- Northern Ireland Chest Heart and Stroke (NICHS) staff
  - Currently working for NICHS as a care coordinator role or PREP delivery physiotherapist
  - Minimum experience of 12 weeks of involvement in PREP
- Other professionals
  - Have experience supporting people with stroke to complete PA within a community setting, for example, exercise, health, voluntary, and statutory organization staff (there is no minimum amount of experience required—ie, even if they have supported one person, they can still participate—and therefore, there is no additional criteria required for these participants).

Exclusion criteria (NICHS staff and other professionals)

- Experience of supporting people with stroke to complete PA within the acute hospital setting only
- Unable to use/access a digital device with internet access and a microphone (for online focus group only)
- Being a member of the advisory board

### Recruitment

PREP participants and their carers will be told about the research study through their care coordinators at their PREP group. A member of the research team will visit the PREP group to distribute the information sheet. The researcher will obtain written consent from the interested participants and collect additional information: demographic information, date of stroke, and self-reported pre- and poststroke activity level. Contact information will be gathered so participants can be contacted for the online focus group or further stages of the project. PREP staff will be contacted through a list provided by one of the advisory board members who oversees the PREP groups. Other professionals will be contacted using flyers posted on social media and through previous study contacts. The research associate will then make contact to discuss the project. Interested individuals will be emailed the participant information sheet and an electronic copy of the consent form. Participants will be contacted if they are interested but cannot participate in one of the focus groups. Participants are free to withdraw from participation at any stage; however, all data collected up to the point of withdrawal will be retained in the study. If they provide a reason for why they withdraw or cannot continue on to the next stages, the reasons will be documented.

### Stakeholder Consultation Procedure (Focus Groups)

Two focus groups of PREP participants will take place, with a goal of including 4 to 8 participants in each group. This will involve participants who have completed at least 6 weeks of the PREP program, including both current and previous participants who are now volunteers. We also aim to recruit carers to provide feedback and insights in the participant focus group. One focus group will occur in person and one online, unless the majority of participants prefer one method. This will allow for a wider range of participants from across Northern Ireland to provide feedback. Two professional focus groups will also take place. For the first one, a PREP staff focus group (4-8 participants) will occur. The PREP staff are made up of care coordinators and physiotherapists who work on a weekly basis within NICHS to run the PREP groups. The last focus group will consist of other professionals: health care trust physiotherapists, personal trainers, and any other exercise professional with experience working with survivors of stroke. All focus groups will be conducted by the research associate and a cofacilitator from the research team, will be audio-video recorded, and are expected to last 1 hour. A semistructured topic guide will be used based on the scoping review analysis. After each focus group, observations will be typed, and the transcript will be generated from the built-in transcription software in Microsoft Teams (version 1416/1.0.0.2026062402/0326). This transcription will then be verified by the research associate.

### Co-Production (Stage 2)

Stage 2 aims to coproduce the content, structure, and materials of the PREP maintenance program, building on stage 1 data. The implementation framework [45] provides a guide for examining situational factors, feasibility, acceptability, and the delivery of group discussions. This framework will help structure the workshop discussions in the second stage. A list of prompts based on the framework sections will be used by the research associate facilitating the group. Participants who complete stage 1 will be informed at the end of the focus group about the stage 2 online co-production workshops and invited to participate. We are aiming to recruit 2 participants from each stakeholder group (ie, physiotherapists, exercise professionals, statutory/voluntary organization staff, survivors of stroke, and their carers; n=6-10). Interested participants will be contacted and sent relevant information via email. A waitlist will be created in case group participants drop out. If additional participants (professionals) are required, social media recruitment will occur using the recruitment flyer as per stage 1. If additional survivors of stroke are required, the recruitment methods described in stage 1 will be used. Based on the TIDieR (Template for Intervention Description and Replication) framework [46], three workshops will take place online. Workshop one will focus on intervention content and workshop two on intervention structure such as dose and outcome measurements. The final workshop will focus on intervention materials.

Within each 90-minute session, discussions will be cofacilitated by a researcher and service user from the advisory group to ensure each participant has an equal voice [47]. Facilitators will encourage a group decision on the priority of the ideas using a consensus approach [47]; if consensus cannot be gained, the views of those with lived experience will be prioritized. Electronic data will be captured during workshops via various methods such as whiteboards and polls on Microsoft Teams. The workshops will also be audio-video recorded to support the research associate with data collection and analysis. Between workshops, the research associate will review the information and share with participants via email. Participants will be asked to read the information before attending the subsequent workshop.

### Prototype Development (Stage 3)

The developed intervention will be shared with the project advisory group to receive expert review from the advisory board as per recommendations within the DECIPHer framework [36]. The individuals on the advisory board are staff members from NICHS who are focused on the stroke population within the charity, an individual who has survived a stroke and completed PREP, and other researchers across the island of Ireland. This discussion with the advisory board will help fine-tune the intervention from the proposed formats from the workshops. Next, the proposed intervention will be discussed with the research group to finalize the future program materials and contents.

### Data Analysis

Qualitative data collected via audio recordings will be transcribed verbatim by the research associate where required (eg, focus groups) and analyzed using reflexive thematic analysis [48] with NVivo 15 software (Lumivero). This form of data analysis involves collecting data, familiarization with the dataset through reading and rereading, generation of initial codes, combining codes according to shared meaning to generate themes by one researcher, and reviewing potential themes by the research team to define and name the final themes.

The analysis of the focus groups in stage 1 will be used to create topic guides for the workshops in stage 2. The results from the workshops will then be used to create the initial iteration of the PA maintenance program.

### Risks to Participants

Risks to participants and researchers are considered minimal. A distress protocol is in place should any participant become emotionally upset during the study. In the occurrence of any adverse events, a record will be kept of all information on the instances, including mental and physical health problems.

### Ethical Considerations

Ethics approval for this study was obtained in October 2024. This study has been approved by Ulster University (REC/24/0044). Those participating in the study will be informed of the purpose, data collection, and handling procedures.

All participants taking part in the study will be assigned a code to protect their identity. The information sheet provided to the participants will explain how their data is collected and how the confidentiality of their data is kept. Any paper copies of data will be kept in a locked cabinet, while any electronic copies will be password protected and stored securely on the university server to retain confidentiality. Data monitoring will be completed by the advisory and stakeholder groups, depending on the stage. An internal audit will be completed by the research team each quarter. Individuals on the advisory board will be compensated for their time in the form of a voucher as well as being named as an author for any publications.

### Dissemination Plans

Any results from the study will be published in a peer-reviewed journal and the university data repository upon the conclusion of the study. The project advisory board will discuss audiences to target for dissemination once results have been generated. Dissemination will occur when the study has completed.

## Results

A total of five focus groups were completed (N=38 total participants) over a span of 3 months (September to November 2025). Two main themes developed from the focus groups: shifting realities of PA post stroke and motivation through community. These themes and analyses were used to create topic guides for the workshops in stage 2.

## Discussion

**Figure 1.**
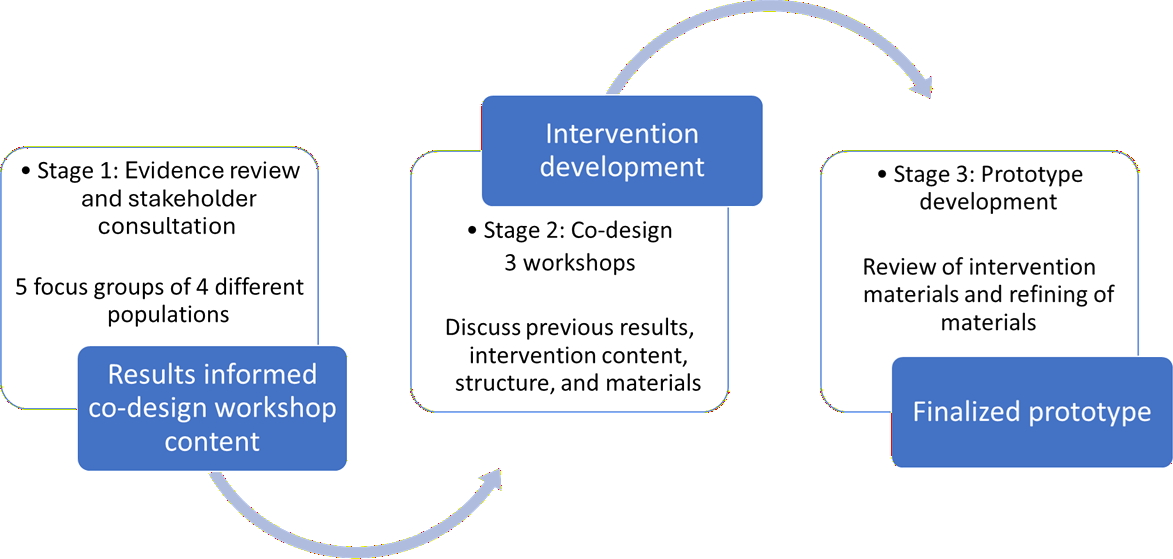
The three stages of the Centre for Development, Evaluation, Complexity and Implementation in Public Health Improvement (DECIPHer) framework and key parts of the stages.

We hypothesize that the feedback from the focus groups will provide us with deeper insights into tailoring a PA program for survivors of stroke. Previous literature has shown the barriers that an individual may encounter post stroke when trying to engage in PA along with the barriers an exercise professional may encounter [49-51]. The DECIPHer co-production framework was chosen as the most appropriate to guide an intervention development that was anchored on working with survivors of strokes to design the intervention. The three stages of the DECIPHer framework, seen in Figure 1, allow for everyone involved to share their views while working toward developing an intervention pilot during the focus groups and workshops. Once the first two stages are completed, a third stage will allow for the finalization of these views to develop an intervention to pilot. For the workshops, we will use the TIDieR framework to provide a checklist of items to cover in the workshops to assess the feasibility of the planned intervention [46]. Both of these frameworks provide a clear outline for the intervention development [36,46] and have been used previously to guide exercise interventions involving different stakeholders [36,46,52,53]. One potential limitation is that participants will be recruited from the current PREP classes and not stroke survivors who are not currently engaging in any form of PA. While experience-based co-design [54] provides rich contextual insights, it does not offer sufficient structure for specifying, standardizing, and reporting intervention components for replication and evaluation. Therefore, DICEPHer, supported by the TIDieR checklist, was selected to ensure transparency, reproducibility, and scalability. This protocol outlines the different stages of the research study that will take place to create a PA maintenance intervention program for survivors of stroke. Once the data is finalized and analyzed, the pilot will begin.

### Strengths and Limitations

One strength of this study is that we have included a wide range of stakeholders in the development of this study and have included their feedback through each of the stages. Another strength is that the pilot intervention will occur across the country in three different health trusts to allow for a mixture of participants. By doing this, we will have older trained and newly trained exercise professionals taking part in the intervention. However, a limitation is that the newly trained individuals may not have any experience working with survivors of stroke and may not be fully aware of their abilities and barriers. Additionally, while this population is representative of people living with stroke in Northern Ireland, participants are being recruited based on their completion of PREP. Therefore, the views of people living with stroke who have not completed PREP are not included in this study.

### Conclusions

This co-design study aims to develop an intervention for a community-based PA program. By involving different stakeholders, the intervention will be tailored toward those who have survived a stroke and those who are trained in exercising that population. This will provide a deeper understanding of the barriers to PA that survivors of stroke face on a daily basis as well as the type of engagement needed for maintaining their PA. We have allowed for three waves of the pilot to occur, which will allow for any intervention changes to be made based on feedback from those involved in the previous wave. This will allow us to create an ideal PA maintenance program for this population.

## Abbreviations

DECIPHer: Centre for Development, Evaluation, Complexity and Implementation in Public Health Improvement
NICHS: Northern Ireland Chest Heart and Stroke
PA: physical activity
PREP: Post Rehabilitation Exercise Program
TIDieR: Template for Intervention Description and Replication
